# Transformation of arsenic species from seafood consumption during *in vitro* digestion

**DOI:** 10.3389/fnut.2023.1207732

**Published:** 2023-10-12

**Authors:** Bei Liu, Jianxin Sui, Ruixue Feng, Hong Lin, Xiangning Han, Xun Sun, Limin Cao

**Affiliations:** Food Safety Laboratory, College of Food Science and Engineering, Ocean University of China, Qingdao, China

**Keywords:** arsenic species, seafood, *in vitro* digestion, transformation, risk assessment

## Abstract

Arsenic (As) species analysis is important for the risk evaluation of seafood. Until now, there has been limited information on the change of As species during digestion. Here, the As species in different types of seafood before and after *in vitro* digestion were investigated. Although inorganic As was not detected in digested fish samples, As(V) contents in digested crabs and scallops were 17.12 ± 1.76 and 138.69 ± 7.53, respectively, which were approximately 2–3 times greater than those of the pre-digestion samples. In further experiments, arsenocholine, dimethylarsinate, arsenobetaine, and monomethylarsonate were all convertible to As(V) during *in vitro* digestions with different rates. The transformation demonstrates a complex process and could be affected by many factors, such as pH, time, and digestion juice composition, of which pH seemed to be particularly important. Free radicals were responsible for the oxidation in the transformation reactions. Unlike arsenobetaine, arsenocholine seemed to be able to directly transform to monomethylarsonate without the intermediate dimethylarsinate. This study reveals and validates the potential of other species (oAs or/and unknown species) to convert to iAs, identifies the main factors affecting this process, and proposes a reaction pathway. There is an important implication for promoting a more accurate risk assessment of arsenic in foodstuffs.

## 1. Introduction

Arsenic (As) is widely present in nature in the form of organic As (oAs) and inorganic As (iAs) (1). Human As exposure typically occurs through oral ingestion, skin contact, and respiratory ingestion, among which oral ingestion of food is the most important (2). Seafood is usually rich in As and is now considered the main source of As exposure for humans (3). Until now, the oAs in fishes, shellfishes, and crustaceans has been identified mainly as arsenocholine (AsC), arsenobetaine (AsB), dimethylarsinate (DMA), and monomethylarsonate (MMA), while iAs usually present as arsenious acid [As(III)] and arsenic acid [As(V)] (4). OAs is traditionally considered as not significantly hazardous but some recent studies have indicated that MMA and DMA, as well as some metabolites of oAs, may be also highly poisonous (5). IAs has been verified to exhibit strong biotoxicity (4). For example, As(III) interferes with enzyme catalysis in the human body and leads to slow cell decay, and As(V) competes with phosphate and disrupts the production of adenosine triphosphate (ATP) (6). Therefore, iAs is now used to assess the risk of As, and the maximal residue level (MRL) has been established in China, the European Union, the United States, and other countries (7–9). For the risk assessment of chemical hazards in foodstuffs, the bio-accessibility analysis is gaining increasing attention. After oral intake, these chemicals are transferred to the gastrointestinal tract; however, only a proportion of them can be absorbed by the human body and cause toxic effects. Information on the real absorption of different As species in the human gastrointestinal tract is very limited, but according to some studies with mice, AsB has a significantly higher absorption efficiency (over 96%) than As(III) and As(V) (approximately 81 and 86%, respectively), and significant biotransformation of AsB to [As(V)] following oral administration has been indicated (10). Therefore, fully considering bio-accessibility (the proportion of these chemicals that have undergone possible biotransformation during digestion and can be released from the food matrix for human body absorption) in toxicity evaluation seems to be more accurate than just analyzing the undigested food sample itself. It can not only reflect the influence of gastrointestinal conditions on the physicochemical properties of the chemicals but also indicate the possible interconversion among different species. So far, many *in vitro* methods have been developed for this purpose, such as the physiologically based extraction test (PBET), *in vitro* gastrointestinal (IVG) method, and European Unified Bio-accessibility Research Group (BARGE) method (UBM) (11–13), of which the UBM method is widely recognized and used for different environmental and food samples (14–16). Now there is also an emerging trend to develop dynamic models to simulate more effectively the complex human gastrointestinal tract, such as the regular secretion of digestion juice, the dynamic pH values, and the influence of gut microbes (17, 18).

Previous studies have also found that the bio-accessibility of iAs in foodstuffs can be elevated during digestion. For example, Chavez-Capilla et al. evaluated the bio-accessibility of As in some food samples (rice, seaweed, shellfish, etc.) and found a significantly increased iAs content after *in vitro* digestion, suggesting that oAs had been converted to the more hazardous iAs during digestion (19–21). Wiele et al. (22) found that human colonic microbes also have the potential to actively metabolize arsenic into methylated arsenate and thioarsenate. Some chemical reactions can also mediate the interconversion among different As species, e.g., water-soluble compounds (such as glutathione) with antioxidant capacity in vegetables have the reducing activity to convert pentavalent arsenic into trivalent arsenic (23) and induce the demethylation of DMA (20). In addition, oxidative conversion has been studied by Chávez-Capilla and coworkers, who found that arsenosugars could be converted to inorganic arsenic species in simulated gastric juices (19). These results provide important clues that the content of iAs in foods may become higher after digestion, which increases the bio-accessibility of iAs, potentially resulting in higher toxicity. However, detailed and convincing information about the bio-accessibility and interconversion among different As species in food is still very limited, especially in seafood.

To help solve the question, here, the bio-accessibility of oAs (AsB, AsC, DMA, and MMA) and iAs [As(III) and As(V)] in some seafood samples (swimming crab, *Portunus trituberculatus*; scallop, *Azumapecten farreri*; turbot, *Scophthalmus maximus*; and flounder, *Paralichthys olivaceus*) was investigated using the UBM method. Then, the potential of other species (oAs or/and unknown ones) to transform to iAs was revealed, the main factors that influence the process were identified, and the reaction pathway was proposed. These findings contribute to a broader comprehension of the potential hazards associated with arsenic in seafood. They help to offer a more objective and scientific assessment of the health risks posed by heavy metal contamination in foodstuffs.

## 2. Materials and methods

### 2.1. Chemicals and samples

Ultrapure Water (purified by a Milli-Q system, 18 MΩ cm, Millipore-Q, Burlington, MA, United States) was used to prepare all solutions. Nitric acid (65%, v/v, GR grade) was used for the wet digestion of food samples and extraction of different As species. Standard solutions of AsC (0.374 μmol·g^−1^, GBW08671), AsB (0.518 μmol·g^−1^, GBW08670), DMA (0.706 μmol·g^−1^, GBW08669), MMA (0.335 μmol·g^−1^, GBW08668), As(III) (1.011 μmol·g^−1^, GBW08666), and As(V) (0.233 μmol·g^−1^, GBW08667) were purchased from the National Standards Substance Center (Beijing, China).

Commercial grade swimming crab was obtained from Qingdao West Coast New Area, scallop was obtained from Muping District, Yantai City, and turbot and flounder were obtained from Nanshan Market of Qingdao City in China. The crude samples were fully washed with water and steamed for a certain time according to a Chinese consumption custom (swimming crab was placed into a pot with hot water and steamed for approximately 20 min, and the body completely turned red; scallop was placed in boiling water for 5 min; and turbot and flounder were placed in boiling water for 10 min). Then the edible tissues of each kind of sample were dissected out, combined, and homogenized with a small meat mincer, packed in plastic valve bags with detailed markers, and stored at −40°C for further use.

### 2.2. Methods

#### 2.2.1. *In vitro* digestion

The *in vitro* digestion was performed referring to the UBM method described by Wragg et al. (13). The digestion juice contents are summarized in Supplementary Tables 1–4. Briefly, 0.5 g of food samples (denoted as pre-digestion sample, PDS) were placed into 100 mL sample bottles. At first, 7.5 mL of saliva was added and the samples stood for 5 min. Then, 11.5 mL of gastric juice was added and the samples were shaken at 100 rpm for 60 min at 37°C (Constant Shaking Incubator, ZWY-2102C, Zhicheng). Then, 22.5 mL of intestinal juice and 7.5 mL of bile were added and shaken at 100 rpm for 4 h at 37°C. The digestion of food samples was performed using a NutriScan GI 20 Glycemic Index Analyzer at the College of Food Science, Qingdao Agricultural University. At the end of the gastric (G) phase and gastrointestinal (GI) phase, 19 and 49 mL of digestive samples were collected, respectively, and immediately cooled at −20°C for 10–15 min to stop the enzymatic reactions (13). Then, the digestive samples were centrifuged at 8,778 × *g* at 25°C for 10 min (High Speed Centrifuge, Neo 15, Heal Force), and the supernatants were collected and filtered through a 0.22 μm Cellulose Acetate (CA) membrane (Membrane, China) to obtain the digested samples (DS) for the analysis of As content. The *in vitro* digestion of standards was performed similarly, except that the seafood sample was replaced with a certain amount of arsenic standard solution to reach the required concentration (Supplementary Table 5). The effect of the radical scavengers was evaluated in the *in vitro* gastric digestion of AsB. Radical scavengers (a mixture of 1 mL of 0.1 μg·ml^−1^ vitamin C solution, 1 mL of 0.1 μg ml^−1^ catechin solution, and 1 mL of 0.1 μg ml^−1^ cysteine solution) were added to the gastric juice containing AsB and then reacted in argon for 1 h. The digestion juice as the blank demonstrated no significant interference to the high-performance liquid chromatography with inductively coupled mass spectrometry (HPLC-ICP-MS) analysis of As species in digested samples (Supplementary Figure 1).

#### 2.2.2. Determination of As content

The total As (tAs) in both PDS and DS were determined by inductively coupled plasma mass spectrometry (ICP-MS), as previously described, in which the reliability of the technique was verified by analyzing certified reference materials GBW 10024 (scallop, Institute of Geophysical and Geochemical Exploration) and SRM 1566b (oyster tissue, National Institute of Standards and Technology) (24). Briefly, 1.0 g of PDS or 5 mL of DS were placed into quartz digestion tubes, mixed with 4 mL of HNO_3_ and 1 mL of HClO_4_, and incubated overnight at room temperature. The mixture was heated at 140°C for 2 h, followed by heating up to 185°C until approximately 1 mL of solution remained in a digestion system (EHD-24). Then, the solution was transferred to microcentrifuge tubes, where the volume was maintained at a constant level of 5 mL with ultra-pure water. The samples were filtered through a 0.22 μm membrane and analyzed by inductively coupled plasma-mass spectrometry (ICP-MS, Agilent 8800, United States). Three parallel experiments were conducted for each sample. A blank was prepared according to the same operation without the addition of samples.

Different As species were determined by HPLC-ICP-MS (1260 Infinity HPLC, Agilent, United States; ICP-MS, Agilent 8800, United States) according to the method described by Chen (25), which can effectively separate and detect six As species (Supplementary Figure 2). DS was not treated, and PDS was pretreated by HNO_3_ according to the following method: in brief, 1.0 g of PDS was put in a 50 mL centrifuge tube and mixed with 20 mL of 1% HNO_3_ solution. The mixture was incubated at 90°C for 2.5 h, and during the incubation, the sample was shaken for 1 min every 0.5 h. After cooling to room temperature, the solution was centrifuged at 6,225 × *g* at 25°C for 15 min and filtered through a 0.22 μm membrane. The crab paste and crab roe were defatted with hexane after centrifugation: 5 mL of supernatant was added to a 50 mL centrifuge tube, followed by 5 mL of hexane, and then the solution was shaken for 1 min. Then, the mixture was centrifuged at 6,225 × *g* for 15 min, the upper hexane layer was discarded, and the remaining solution was treated with hexane again according to the same procedure. Finally, the aqueous layer was collected and filtered through a 0.22 μm membrane. Three parallel experiments were conducted for each sample. A blank was prepared according to the same operation without the addition of samples. The instrumental conditions for ICP-MS and HPLC-ICP-MS are summarized in Supplementary Table 6.

#### 2.2.3. Statistical analyses

Bio-accessibility was calculated according to the following formula (26):


$$\text{Bio-accessibility }(\%)=\left(C_{IV}\times V_{IV}\right)/\left(T_{S}\times M_{S}\right),$$

where C*_IV_* is the As concentration (μg·ml^−1^) in DS, V*_IV_* is the volume of the DS (ml), T*_S_* is the As concentration in PDS (μg·g^−1^), and M*_S_* is the mass of PDS (g).

Differences among different groups were tested by Student’s *t*-test, in which the significant difference was considered as *p* < 0.05. Unless otherwise stated, the results were analyzed based on triplicates, and all data were expressed as mean ± standard deviation (SD).

## 3. Results and discussion

### 3.1. Content of different As species in the four seafoods

The tAs content of the PDS was found within the range from 1.8 to 8.3 μg·g^−1^, with the highest content in swimming crab and the lowest in flounder (Table 1). Similar to previous studies (3, 4), oAs (mainly AsB) was evaluated over 75–90% of the tAs. Toxic As(III) and As(V) were detected in all samples but the concentrations were far lower than those of oAs. The tAs was approximately 10–25% higher than the sum of six different forms of arsenic, and the difference should be due to some unknown As species, which could not be fully extracted or/and could not be identified with the available techniques. After the *in vitro* digestion, the As content changed significantly in all samples (Table 2). Owing to the possible loss by precipitation, complexation, and other physical/chemical interactions, the bio-accessibility of the target is usually considered below 100% (27). However, here the content of As(V) in crabs and scallops was found to be 17.12–138.69 ng·g^−1^, which was approximately 2–3 times greater than those in the PDSs of the same samples (Table 1). Similarly, significant increases in iAs during digestion were also reported by previous studies on shellfish, rice, seaweed, etc. (19, 20). These results suggest that the real risk of As in these foodstuffs might have been underestimated if the bio-accessibility was not considered (28–30). Notably, for the two fish samples, iAs was not detected after *in vitro* digestion but the AsB content significantly increased (*p* < 0.05). This trend was totally different from that of crabs and scallops, indicating that the change of As species in fish samples might be significantly different from that of other seafood.

**Table 1:** Contents of different As species (ng·g^−1^) in pre-digestion samples (*n* = 3).

Samples		tAs	AsB	AsC	DMA	MMA	As(III)	As(V)
Swimming crab	Muscle	6042.67 ± 179.72	4388.99 ± 155.28	69.68 ± 35.68	311.53 ± 75.60	< DL	4.17 ± 0.00	7.52 ± 0.89
	Crab Paste	4234.76 ± 902.12	3515.81 ± 711.20	50.45 ± 10.08	231.33 ± 57.01	23.45 ± 1.36	5.59 ± 1.00	8.26 ± 1.36
	Crab roe	5816.90 ± 684.93	4294.83 ± 817.52	49.98 ± 30.19	165.07 ± 19.90	29.25 ± 4.18	6.30 ± 0.34	9.92 ± 0.75
Scallop		1892.26 ± 134.48	1583.09 ± 147.02	208.13 ± 4.05	24.80 ± 0.70	48.09 ± 0.15	8.13 ± 0.69	67.79 ± 3.03
Turbot		3821.17 ± 89.99	2996.16 ± 98.72	91.13 ± 4.100	78.21 ± 3.68	< DL	< DL	88.72 ± 6.26
Flounder		1798.99 ± 77.45	1607.02 ± 380.81	111.78 ± 16.60	94.84 ± 6.59	< DL	< DL	111.7 ± 15.59

DL, the lowest detected level.

**Table 2:** Content of As (ng·g^−1^) in the digested seafood samples (*n* = 3).

Samples			tAs	AsC	AsB	DMA	MMA	As(III)	As(V)
Swimming crab	Muscle	G	4078.90* ± 239.08	< DL	2943.99* ± 249.83	89.73* ± 13.65	< DL	1.98* ± 0.07	17.12* ± 1.76
		GI	5435.43* ± 53.78	< DL	4085.57* ±154.67	150.44* ± 27.27	< DL	< DL	24.57* ± 2.63
	Crab Paste	G	2532.07* ± 168.87	< DL	2115.23* ± 246.66	80.47* ± 11.13	10.36* ± 1.24	1.89* ± 0.15	22.26* ± 1.79
		GI	3754.97 ± 83.02	< DL	3033.31 ± 348.75	76.05* ± 12.10	< DL	< DL	26.73* ± 0.91
	Crab roe	G	4045.87* ± 177.16	< DL	2180.68* ± 54.09	112.85* ± 12.69	19.92* ± 3.38	2.19* ± 0.34	20.83* ± 2.32
		GI	4799.51* ± 6.66	< DL	3324.44 ± 343.43	86.95* ± 8.23	< DL	< DL	28.24* ± 2.21
Scallop		G	1241.61* ± 126.06	134.22* ± 4.50	1091.89* ± 162.09	14.79* ± 2.26	32.23* ± 1.81	< DL	94.65* ± 3.48
		GI	1450.79* ± 221.32	106.79* ± 14.80	1372.36* ± 21.00	12.62* ± 0.88	28.96* ± 0.32	< DL	138.69* ± 7.53
Turbot		G	2540.97* ± 151.44	58.85* ± 0.40	3687.15* ± 93.24	< DL	< DL	< DL	< DL
		GI	2746.91* ± 420.30	< DL	5549.26* ± 237.18	< DL	< DL	< DL	< DL
Flounder		G	1483.27* ± 64.58	51.61* ± 1.53	2121.13* ± 84.08	< DL	< DL	< DL	< DL
		GI	1547.93* ± 221.70	< DL	3132.90* ± 190.39	< DL	< DL	< DL	< DL

DL, the lowest detection limit. The asterisk (*) indicates that there is a significant difference between the sample after digestion and the pre-digestion sample (Table 1) with Student’s *t*-test (*p* < 0.05).

In principle the increased As(V) bio-accessibility could be attributed to the following reasons:

1. Release of bound-As species: for the extraction of As species, the PDSs were treated with HNO_3_ and heated at 90°C for 2.5 h. Some previous studies have indicated the affinity of oAs and iAs with proteins or peptides, and the interaction was demonstrated to be unstable at high temperatures in the presence of strong acids (31–33). Therefore, such an HNO_3_ pretreatment may effectively degrade the protein-based complex in the PDS and therefore release the bound chemicals (34), but it seems very difficult to elucidate the dissociation rate and the amount of remaining bound-As complex. Considering that the extraction recovery of tAs was only approximately 75%, there was still a significant amount of As compounds unable to be effectively extracted and detected in PDS, and they may be effectively released under the digestion conditions in the form of detectable As(V) (with or without chemical transformation), therefore resulting in the increase in bio-accessibility. Additionally, some experiments using food samples have proposed the release of bound-As during *in vitro* digestions (20).
2. Direct transformation of free oAs or unknown As species to As(V): when further validated with AsB and DMA standards, significant As(V) was observed after the *in vitro* digestion (*p* < 0.05), which clearly confirmed the possible transformation among different As species, especially from oAs to iAs (Table 3). Moreover, the rate of such interconversion varied significantly among seafood samples. This result was in good agreement with Table 2. In crab and scallop, the spiked AsB and DMA also effectively transformed; in particular, the transformation rate of As(V) was even higher than that of the standards.

**Table 3:** The content of As species in different seafoods (spiked with AsB and DMA) after *in vitro* gastric digestion.

Spiking concentration	AsB (421.35)	DMA (108.70)	AsV (0.00)
AsB and DMA standards	389.12* ± 5.87	91.64* ± 0.87	30.82* ± 1.35
Swimming crab (muscle)	381.85* ± 25.82	84.67* ± 9.75	36.28* ± 4.88
Scallop	386.48* ± 25.82	87.77* ± 7.50	35.01* ± 3.72
Turbot	415.76* ± 9.40	106.81* ± 2.87	< DL

DL, the lowest detection limit. The asterisk (*) indicates that there is a significant difference between the sample after digestion and the pre-digestion sample (Table 1) with Student’s *t*-test (*p* < 0.05). The background was tared (calculated for As, ng; *n* = 3).

On the other hand, no interconversion was observed in fish samples, which again showed a different trend from other samples. The result indicates that the fish matrix may have a unique impact on the interconversion pathways of different As species. Similar to this, Chavez-Capilla et al. (19) demonstrated that the dimethylarsinoylpropionic acid in fish samples does not significantly change during gastric and intestinal digestions. However, until now, the unique characteristics of As species in fish samples has not been paid full attention to, and more detailed investigation would be an interesting study.

Owing to the uncertainty of the solubility/stability of different As species during the *in vitro* digestion (35), along with their possible interaction with other components, it seemed very difficult to pin down the contribution of each As species to the increased iAs in the seafood samples. However, the transformation seemed to have occurred during both the G and GI phases, and the increase of As(V) during the G phase was higher than that during the GI phase (Table 4). These details have indicated the importance of G digestion for the proposed release or transformation to oAs.

**Table 4:** Content of As (ng·g^−1^) in the supernatants of gastric-digested muscle of swimming crab and turbot and in supernatants further treated with gastrointestinal digestion (*n* = 3).

Samples		tAs	AsC	AsB	DMA	MMA	As(III)	As(V)
Swimming crab (muscle)	G	4110.46* ± 40.87	< DL	2694.88* ± 87.22	128.88* ± 4.38	19.11* ± 0.00	0.02* ± 1.26	26.75* ± 0.02
	GI	4134.25* ± 10.75	< DL	2639.99* ± 159.71	98.47* ± 1.56	< DL	< DL	38.62* ± 0.06
Turbot	G	2574.47* ± 176.90	13.74* ± 0.10	2495.60* ±73.44	< DL	< DL	< DL	< DL
	GI	2602.69* ± 119.09	< DL	2496.54* ± 26.15	< DL	< DL	< DL	< DL

DL, the lowest detection limit. The asterisk (*) indicates that there is a significant difference between the sample after digestion and the pre-digestion sample (Table 1) with Student’s *t*-test (*p* < 0.05).

### 3.2. Transformation tendency

Various reports have confirmed that the As in seafood is predominately oAs (36), and their potential to transform to As(V) seemed of greater importance. Although several studies have proposed the interconversion by demethylation or other pathways (20), there is still a vacancy for detailed information on the transformation mechanisms among different As species. Therefore, in this study, the possibility and rate of different oAs to transform to iAs were investigated through the *in vitro* digestion of standards (with the same concentration of seafood samples). Such a proposal was confirmed by the significant difference in the peak area of the As species standard before and after *in vitro* digestions (Supplementary Figure 3). As(V) was detected in digested AsC, DMA, and MMA samples, among which the transformation rate of MMA was highest, followed by that of AsC and DMA (Table 5). Similar to experiments with food samples, the G phase contributed most of the increase of As(V), while the increase was much smaller during the GI phase (Supplementary Table 8). AsB has been considered stable enough to endure common chemical and biological treatments (37), and its transformation is usually demonstrated under relatively harsh conditions, such as photo-oxidation or a long reaction time (38, 39). The transformation of AsB to DMA during *in vitro* digestion was observed at a rate of approximately 5.5%. Significant conversion to As(V) was also detected when investigated with a higher concentration of AsB (Table 5). The conversion rate of MMA was the highest, followed by DMA, AsC, and AsB. The conversion rate of AsB was the lowest, which may be determined by the structure of the compound. The simpler the structure, the higher the conversion rate. Considering the abundance of AsB in some seafoods (accounting for more than 70% in PDSs here) and the potential of this compound to be transformed to As(V), the real risk of AsB in these food samples should be more carefully and comprehensively re-evaluated in future studies.

**Table 5:** Transformation of oAs to other As species during *in vitro* digestions (calculated for As, ng; *n* = 3).

Samples		Spiking concentration	AsB	AsC	DMA	MMA	As(III)	As(V)
AsB	G	421.35	388.90 ± 11.38	< DL	11.41 ± 1.63	< DL	< DL	< DL
		1686.39	1500.20 ± 15.23	< DL	116.60 ± 4.01	< DL	< DL	38.04 ± 0.50
	GI	421.35	371.21 ± 21.07	< DL	28.89 ± 0.54	< DL	< DL	< DL
	1% HNO_3_	421.35	406.08 ± 11.10	< DL	8.70 ± 1.17	< DL	< DL	6.66 ± 0.33
AsC	G	45.45	< DL	34.13 ± 0.14	< DL	0.04 ~ 0.16	< DL	6.17 ± 0.69
	GI	45.45	< DL	31.77 ± 2.77	< DL	0.04 ~ 0.16	< DL	7.92 ± 0.42
	1% HNO_3_	45.45	< DL	41.47 ± 1.70	< DL	0.04 ~ 0.16	< DL	3.10 ± 0.44
DMA	G	108.70	< DL	< DL	84.24 ± 6.96	< DL	< DL	10.35 ± 0.85
		27.17	< DL	< DL	23.54 ± 0.46	< DL	< DL	< DL
	GI	108.70	< DL	< DL	75.56 ± 3.37	< DL	< DL	14.47 ± 1.27
	1% HNO_3_	108.70	< DL	< DL	100.10 ± 4.12	< DL	< DL	7.18 ± 2.37
MMA	G	26.79	< DL	< DL	< DL	16.64 ± 2.49	< DL	6.29 ± 0.74
	GI	26.79	< DL	< DL	< DL	13.70 ± 0.83	< DL	8.08 ± 0.58
	1% HNO_3_	26.79	< DL	< DL	< DL	20.70 ± 1.20	< DL	4.62 ± 0.73

DL, the lowest detection limit. Experiments were performed with standards of different As species.

The influence of time, pH value, and enzyme on the transformation from oAs to iAs was further investigated (Tables 6, 7). Using a DMA standard as the reaction substrate, the pH value was proved to be an important parameter for the reaction: when the pH was above 2.0, no As(V) was detected; on the other hand, at pH 1.2, a significant amount of As(V) was observed in both digestive juice and glycine buffers (Table 7). This pH-dependent reaction was even observed in the 1% HNO_3_ solution (pH 0.8–0.9), which is the actual condition used for As extraction from food samples (Table 5), in which AsB, AsC, DMA, and MMA all exhibited significant transformation to As(V). The transformation seemed to occur rapidly, with a 60% transformation within 30 min in most cases (Table 6). Moreover, the reaction rate in digestion juice was higher than that in glycine buffers, indicating that other components (e.g., enzymes) in the digestion juice may also be responsible for the reaction, and this result was consistent with a previous study (20), in which sodium cholate and pancreatin were suggested as important factors for increasing iAs caused by a demethylation reaction during digestion.

**Table 6:** Transformation rate of the DMA standard to other As species in the G phase (calculated for As, %; *n* = 3).

Digestion conditions		AsB	AsC	DMA	MMA	As(III)	As(V)
pH	Time (h)						
1.2	0.5	< DL	< DL	93.48 ± 0.03	< DL	< DL	6.65 ± 0.42
	1.0	< DL	< DL	97.71 ± 0.11	< DL	< DL	9.40 ± 0.01
2	0.5	< DL	< DL	95.66 ± 1.20	< DL	< DL	< DL
	1.0	< DL	< DL	97.10 ± 0.22	< DL	< DL	< DL
4	0.5	< DL	< DL	98.26 ± 2.17	< DL	< DL	< DL
	1.0	< DL	< DL	93.26 ± 0.33	< DL	< DL	< DL
6	0.5	< DL	< DL	99.68 ± 2.39	< DL	< DL	< DL
	1.0	< DL	< DL	101.09 ± 1.52	< DL	< DL	< DL

DL, the lowest detection limit. Adjusted by 12 M HCl and without pepsin.

**Table 7:** The effect of digestion juice composition on As species transformation after the G and GI phases (calculated for As, ng; glycine buffer, pH 1.2; *n* = 3).

Samples	Pepsin	Solution	Time / h	AsB	AsC	DMA	MMA	As(III)	As(V)
DMA (108.70 ng)	√	Digestive juices	0.5	< DL	< DL	83.16 ± 1.96	< DL	< DL	9.40 ± 0.11
			1.0	< DL	< DL	72.83 ± 1.30	< DL	< DL	13.63 ± 0.95
	**√**	Glycine buffer	0.5	< DL	< DL	90.84 ± 2.85	< DL	< DL	6.30 ± 0.44
			1.0	< DL	< DL	77.75 ± 0.85	< DL	< DL	6.63 ± 0.18
	**×**	Glycine buffer	0.5	< DL	< DL	91.70 ± 1.25	< DL	< DL	3.92 ± 0.30
			1.0	< DL	< DL	85.72 ± 2.21	< DL	< DL	4.98 ± 0.46
MMA (26.79 ng)	**√**	Digestive juices	0.5	< DL	< DL	< DL	18.32 ± 1.88	< DL	6.68 ± 0.55
			1.0	< DL	< DL	< DL	17.04 ± 1.29	< DL	7.18 ± 0.01
	**√**	Glycine buffer	0.5	< DL	< DL	< DL	20.63 ± 1.13	< DL	5.28 ± 0.21
			1.0	< DL	< DL	< DL	20.87 ± 0.13	< DL	5.70 ± 0.61
	**×**	Glycine buffer	0.5	< DL	< DL	< DL	21.14 ± 0.54	< DL	5.10 ± 0.32
			1.0	< DL	< DL	< DL	19.15 ± 0.21	< DL	5.31 ± 0.79
AsC (45.45 ng)	**√**	Digestive juices	0.5	< DL	37.91 ± 0.54	< DL	< DL	< DL	4.96 ± 0.21
			1.0	< DL	37.77 ± 1.55	< DL	< DL	< DL	5.12 ± 0.02
	**√**	Glycine buffer	0.5	< DL	37.18 ± 3.00	< DL	< DL	< DL	4.54 ± 0.05
			1.0	< DL	38.59 ± 1.73	< DL	< DL	< DL	4.60 ± 0.05
	**×**	Glycine buffer	0.5	< DL	38.36 ± 0.55	< DL	< DL	< DL	4.23 ± 0.05
			1.0	< DL	38.00 ± 1.18	< DL	< DL	< DL	4.28 ± 1.16

DL, the lowest detection limit; √, in the presence of pepsin; and ×, without pepsin.

Based on results mentioned above, interconversion among different As species (especially from oAs to iAs) under certain *in vitro* digestion conditions was confirmed, and this was not only consistent with some previous studies of food samples (19–21, 36) but was well supported by recent experiments with mice, in which a significant biotransformation of AsB to As(V) was observed in the gastrointestinal tract following oral administration. On the other hand, the information presented here will increase understanding of the biotransformation among As species in mammals and its influencing factors. However, such an interconversion among As species seems to be a very complex process, and it could be affected by many parameters of the samples and experimental conditions, which indicates that lots of work still needs to be carried out in future to provide more detailed and accurate information. The environmental and culture conditions may influence the accumulation and metabolism of As species, and slight differences in experimental conditions, such as acidity, reaction time, oxygen/light exposure, the digestion juice composition, the As concentration used, and analytical performance, may result in a failure to observe such interconversion during *in vitro* digestion (15, 40–44) or significant differences in the suggested reaction sites (stomach or intestine), pathways, and transformation rates (19–21, 36). Besides further validation with more samples of different species, areas, culturing, and processing conditions, another possible way to solve the task is the fabrication of more reasonable and standardized *in vitro* digestion models, such as the use of dynamic models in place of traditional static ones to simulate human digestion more accurately. In this case, the influence of gut microbes should be given full emphasis, considering that many studies have confirmed the significant As interconversion by these microbes. The research conducted by Wiele et al. demonstrated the potential of human colonic microbiota to actively metabolize arsenic into methylated arsenic species and thioarsenates. Additionally, Chi et al. found that disruption of the gut microbiota can alter the methylation transformation of arsenic (22, 45).

### 3.3. Proposed reaction mechanism

Until now, the detailed mechanism of the transformation among different As species remains unclear, especially for oAs such as AsB and AsC. It is widely accepted that the oxidation of As species follows a radical mechanism, although most of these well-controlled transformations utilize photo and/or catalytical activation for the rapid generation of radicals (46–48). However, the absence of these activation factors does not necessarily quench radical oxidation reactions as reactive oxygen radicals exist ubiquitously. Thus, in the present system, we propose that radical oxidation is also the key step governing the degradation of organoarsenic species. To test this hypothesis, AsB was used as a model and a mixture of radical scavengers was added to the oxidation system (keeping the rest of the parameters unchanged). When the concentration of the radical scavengers was in the same order of magnitude as that of AsB, the result (Table 8) showed a significant suppression in DMA formation, which proved the importance of a radical reaction in the process of oxidation.

**Table 8:** Transformation of the AsB standard to other As species (calculated for As, ng) during *in vitro* gastric digestion with radical scavengers (*n* = 3).

	AsB	AsC	DMA	MMA	As(III)	As (V)
AsB (421.35 ng)	407.11 ± 3.57	< DL	1.19 ± 0.50	< DL	< DL	< DL

DL, the lowest detection limit.

**Figure 1:**
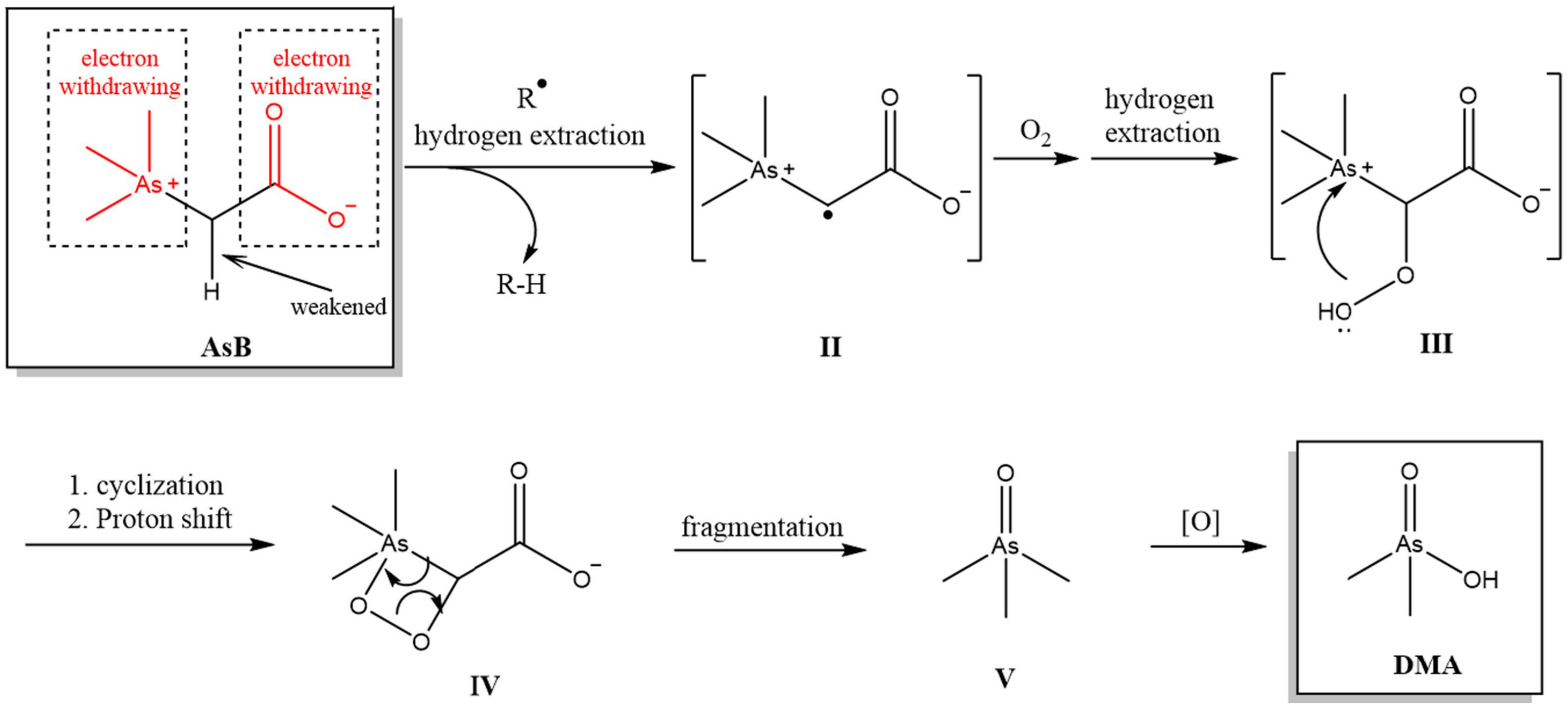
Hypothetical oxidation reaction pathway of AsB.

Another interesting phenomenon was observed during the oxidation of AsB and AsC. The two arsoniums are the major As compounds found in seafood and other foodstuffs, but provide different oxidative products: AsB was oxidized to DMA, whereas AsC yielded MMA. This difference is tentatively explained by different oxidation sequences resulting from structural variation: the oxidation of oAs is a combination of two general types of reaction: the formation of an O-As bond and the breakage of a C-As bond (49). Owing to the different structures of AsB and AsC, the order of these fundamental steps may also be different and result in different key intermediates that yield completely different final products. For example (Figure 1), both the arsonium and carboxylic moieties in AsB are electron withdrawing, which significantly weakens the C-H of the α-H of arsonium through an inducing effect and activates the bond for hydrogen extraction (Figure 1, I- > II) by exiting radicals (R). The resulting radical may be oxygenated (Figure 1, II- > III), then, driven by the high nucleophilicity of peroxide, cyclizes to form a four-membered ring (Figure 1, III- > IV). Owing to the instability of the O-O bond and high ring tension, this 4-membered ring system is prone to fragment (50–52), yielding intermediate V (Figure 1, IV- > V), and the latter could be further oxidized to DMA. On the other hand, the α-H of AsC is less activated as the adjacent CH_2_OH group is generally considered as an electron donating one. Thus, the oxidation might have occurred on As first, and then the C-As bond is cleaved to release MMA. It should be noted that, regarding the difference between the *in vitro* and *in vivo* digestion conditions, the real transformation rate of different As species (and the mechanism involved) in the human body still needs further investigation, in which the influence of food matrix and gut microbes should also be considered.

## 4. Conclusion

In this study, the bio-accessibility of different As species in different seafood samples was determined, and a significant difference was observed. Unlike fish samples, the content of As(V) in digested crabs and scallops was demonstrated to be approximately 2–3 times greater than those of the pre-digestion samples. These results indicated an underestimated risk of these foodstuffs. In further experiments, arsenocholine, dimethylarsinate, arsenobetaine, and monomethylarsonate were all confirmed to be able to convert to As(V) during *in vitro* digestions with different efficiencies. The transformation could be affected by many factors, such as pH, time, and digestion juice, of which low pH seems particularly important. Free radicals were found to be responsible for the oxidation in the transformation reactions. Unlike arsenobetaine, arsenocholine appears to be able to be transformed directly to monomethylarsonate without the intermediate dimethylarsinate. These results provided much new and detailed information on the dynamic changes of As species during seafood digestion, which allowed us to suggest the great scientific significance of a more accurate risk assessment of As in aquatic products and other foodstuffs.

## Data availability statement

The original contributions presented in the study are included in the article/Supplementary material, further inquiries can be directed to the corresponding authors.

## Ethics statement

The studies involving animals were reviewed and approved by the Animal Experimental Ethics Review Committee of School of Food Science and Engineering, Ocean University of China.

## Author contributions

LC, HL, and XS: conceptualization. JS, XH, RF, and BL: experimental design and methodology. BL: formal analysis, investigation, data curation, and writing—original draft preparation. LC and XS: writing—review and editing. LC: funding acquisition. All authors contributed to the article and approved the submitted version.
