# Supplementary material for: Transformation of arsenic species from seafood consumption during in vitro digestion
Source: Front Nutr. 2023 Oct 12;10:1207732. doi: 10.3389/fnut.2023.1207732 (PMC10602890; doi:10.3389/fnut.2023.1207732)
Supplement: Supplementary file 1 [file Data_Sheet_1.docx]

Supplementary Tables

**KCl, NaH_2_PO_4_, KSCN, Na_2_SO_4_, NaCl, NaOH, CaCl_2_, NH_4_Cl, NaCl, NaHCO_3_, KH_2_PO_4,_ MgCl_2_,** **glucose and urea were analytically pure** **chemicals purchased from National Pharmaceutical Group Chemical Reagents Co., Ltd.Glucosamine hydrochloride(G100089), Glucuronic acid(analytically pure), Uric Acid (U105582), Amylase(A109181) and Pancreatin(P110505)** **were purchased from Aladdin Reagent ( Shanghai ) Co., Ltd.**

**Mucin(S12066), Pepsin(S10027), Lipase(S10035) and Bile(S30895) were purchased from Shanghai Yuanye Biotechnology Co., Ltd.**

Supplementary Table 1 The composition of 1000 mL saliva juice

| Reagent | Quantity |
| --- | --- |
| KCl | 896 mg |
| NaH_2_PO_4_ | 888 mg |
| KSCN | 200 mg |
| Na_2_SO_4_ | 570 mg |
| NaCl | 298 mg |
| NaOH (1.0 mol/L) | 1.80 mL |
| Urea | 200 mg |
| Amylase | 145 mg |
| Mucin | 50.0 mg |
| Uric Acid | 15.0 mg |

Supplementary Table 2 The composition of 1000 mL gastric juice

| Reagent | Quantity |
| --- | --- |
| NaCl | 2752 mg |
| NaH_2_PO_4_ | 266 mg |
| KCl | 824 mg |
| CaCl_2_ | 400 mg |
| NH_4_Cl | 306 mg |
| HCl (ω=37%) | 8.3 mL |
| Glucose | 650 mg |
| Glucuronic acid | 20.0 mg |
| Urea | 85.0 mg |
| Glucosaminehydrochloride | 330 mg |
| Bovine Serum Albumin | 1000 mg |
| Mucin | 3000 mg |
| Pepsin | 1000 mg |

Supplementary Table 3 The composition of 1000 mL intestinal juice

| Reagent | Quantity |
| --- | --- |
| NaCl | 7012 mg |
| NaHCO_3_ | 5607 mg |
| KH_2_PO_4_ | 80 mg |
| KCl | 564 mg |
| MgCl_2_ | 50.0 mg |
| HCl (ω=37%) | 180 μL |
| Urea | 100 mg |
| CaCl_2_ | 200 mg |
| Bovine Serum Albumin | 1000 mg |
| Pancreatin | 3000 mg |
| Lipase | 500 mg |

Supplementary Table 4 The composition of 1000 mL bile

| Reagent | Quantity |
| --- | --- |
| NaCl | 5259 mg |
| NaHCO_3_ | 5785 mg |
| KCl | 376 mg |
| HCl (ω=37%) | 180 μL |
| Urea | 250 mg |
| CaCl_2_ | 222 mg |
| Bovine Serum Albumin | 1800 mg |
| Bile | 6000 mg |

Supplementary Table 5 The volume and concentration of As species standards used for the investigation of interconversion during the *in vitro* digestion.

|  | Concentration (μg·mL^−1^ ) | Volume (mL) | Mass (ng) (calculated for As) |
| --- | --- | --- | --- |
| AsB | 1000 | 1 | 421.35 |
|  | 1000 | 4 | 1686.39 |
| AsC | 100 | 1 | 45.45 |
| DMA | 200 | 1 | 108.70 |
|  | 50 | 1 | 27.17 |
| MMA | 50 | 1 | 26.79 |

Supplementary Table 6 Operation conditions for analysis of tAs and As species.

| Parameter |  | | | | Conditions | | | | | |
| --- | --- | --- | --- | --- | --- | --- | --- | --- | --- | --- |
| **HPLC** |  | | | |  | | | | | |
| Analytical column |  | | | | Hamilton PRP-X100 anion-exchange HPLC column  (250 mm × 4.1 mm, 10 μm) | | | | | |
| Mobile phase A |  | | | | Water | | | | | |
| Mobile phase B |  | | | | Methyl alcohol | | | | | |
| Mobile phase C |  | | | | 100 mmol·L^-1^ (NH_4_)_2_CO_3_ in water | | | | | |
| Mobile phase D |  | | | | Methanol（0.06 M HNO_3_）Column sealing liquid | | | | | |
| Injection volume |  | | | | 20 μL | | | | | |
| Column temperature |  | | | | 25℃ | | | | | |
| Gradient program | Time (min) | A% | | B% | | | | C% | | Flow rate (mL·min^-1^) |
|  | 0.00 | 82.0 | | 3.0 | | | | 15.0 | | 1.5 |
|  | 2.00 | 82.0 | | 3.0 | | | | 15.0 | | 1.5 |
|  | 2.50 | 51.0 | | 4.0 | | | | 45.0 | | 1.5 |
|  | 10.00 | 51.0 | | 4.0 | | | | 45.0 | | 1.5 |
| Posttime | 3.00 | 82.0 | | 3.0 | | | | 15.0 | | 1.5 |
| **ICP-MS** |  | | | |  | | | | | |
| Radio frequency power | 1550 W | |  | | |  |  | |  |  |
| Plasma gas flow | 15.0 L·min^-1^ | |  | | |  |  | |  |  |
| Carrier gas flow | 1.01 L·min^-1^ | |  | | |  |  | |  |  |
| Auxiliary gas flow | 1.0 L·min^-1^ | |  | | |  |  | |  |  |
| Peristaltic pump | 0.3 rps | |  | | |  |  | |  |  |
| Nebulizer chamber temperature | 2℃ | |  | | |  |  | |  |  |
| Energy discrimination | -7 V | |  | | |  |  | |  |  |
| Deflect | -5 V | |  | | |  |  | |  |  |
| Q1 bias | 0.0 V | |  | | |  |  | |  |  |
| Q1 prefilter bias | -24 V | |  | | |  |  | |  |  |
| Q1 postfilter bias | -23 V | |  | | |  |  | |  |  |
| Octopole radio frequency | 180 V | |  | | |  |  | |  |  |
| Dwell time | 100 ms | |  | | |  |  | |  |  |

Supplementary Table 7 Peak area of HPLC-ICP-MS for different As species standards before and after *in vitro* digestion

| Samples | | Peak area | | | | |
| --- | --- | --- | --- | --- | --- | --- |
|  |  | AsC | AsB | DMA | MMA | As(Ⅴ) |
| AsC | befor | 362.33 | < DL | < DL | < DL | < DL |
|  | after | 295.57 | < DL | < DL | 19.51 | 74.32 |
| AsB | befor | < DL | 8053.79 | < DL | < DL | < DL |
|  | after | < DL | 6617.15 | 969.85 | < DL | 405.68 |
| DMA | befor | < DL | < DL | 1264.70 | < DL | < DL |
|  | after | < DL | < DL | 1012.86 | < DL | 149.78 |
| MMA | befor | < DL | < DL | < DL | 395.04 | < DL |
|  | after | < DL | < DL | < DL | 332.23 | 85.78 |

*DL: the lowest detected level.

Supplementary Table8 Transformation of DMA standard (108.7 ng) to other As species (calculated for As, ng) during *in vitro* digestion.

| As species | | AsB | AsC | MMA | As(Ⅲ) | As (Ⅴ) |
| --- | --- | --- | --- | --- | --- | --- |
| Phase | Time/h |  |  |  |  |  |
| G | 0.5 | < DL | < DL | < DL | < DL | 6.12 ± 0.42 |
|  | 1 | < DL | < DL | < DL | < DL | 6.76 ± 0.21 |
| GI | 2 | < DL | < DL | < DL | < DL | 9.50 ± 0.74 |
|  | 3 | < DL | < DL | < DL | < DL | 10.14 ± 0.21 |
|  | 4 | < DL | < DL | < DL | < DL | 10.24 ± 0.21 |
|  | 5 | < DL | < DL | < DL | < DL | 10.35 ± 0.11 |

*DL: the lowest detected level.

**Supplementary Figure**

Supplementary Fig. 1. The representative HPLC-ICP-MS result of the *in vitro* digestion blank (performed with only the digestion juice).

Supplementary Fig. 2. The representative of HPLC-ICP-MS chromatogram of arsenic species standards (in order of retention time: AsC, AsB, As(Ⅲ), DMA, MMA, As(Ⅴ). The concentration of standard solution was 100 ng/mL).


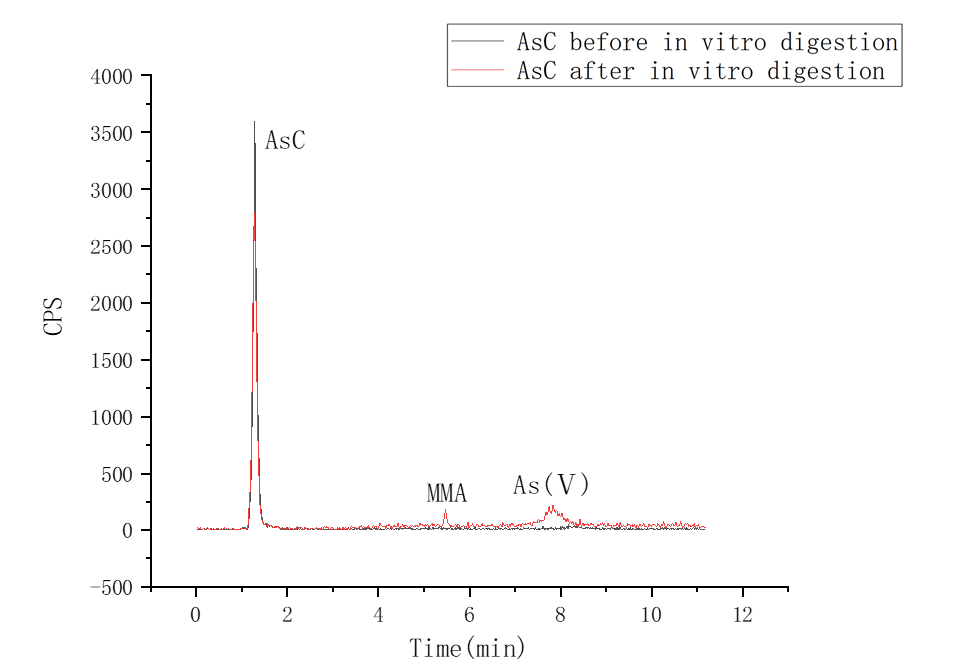


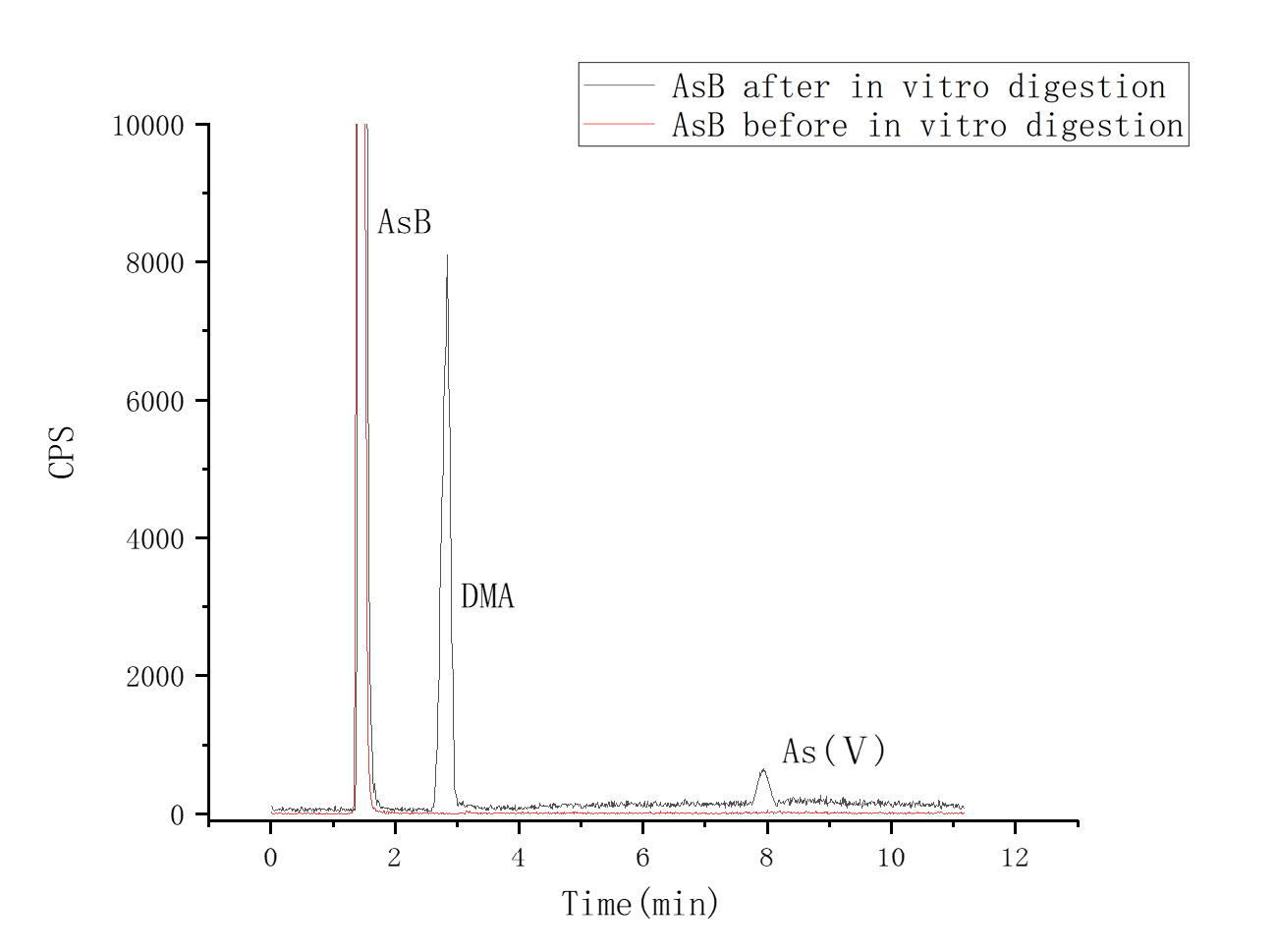


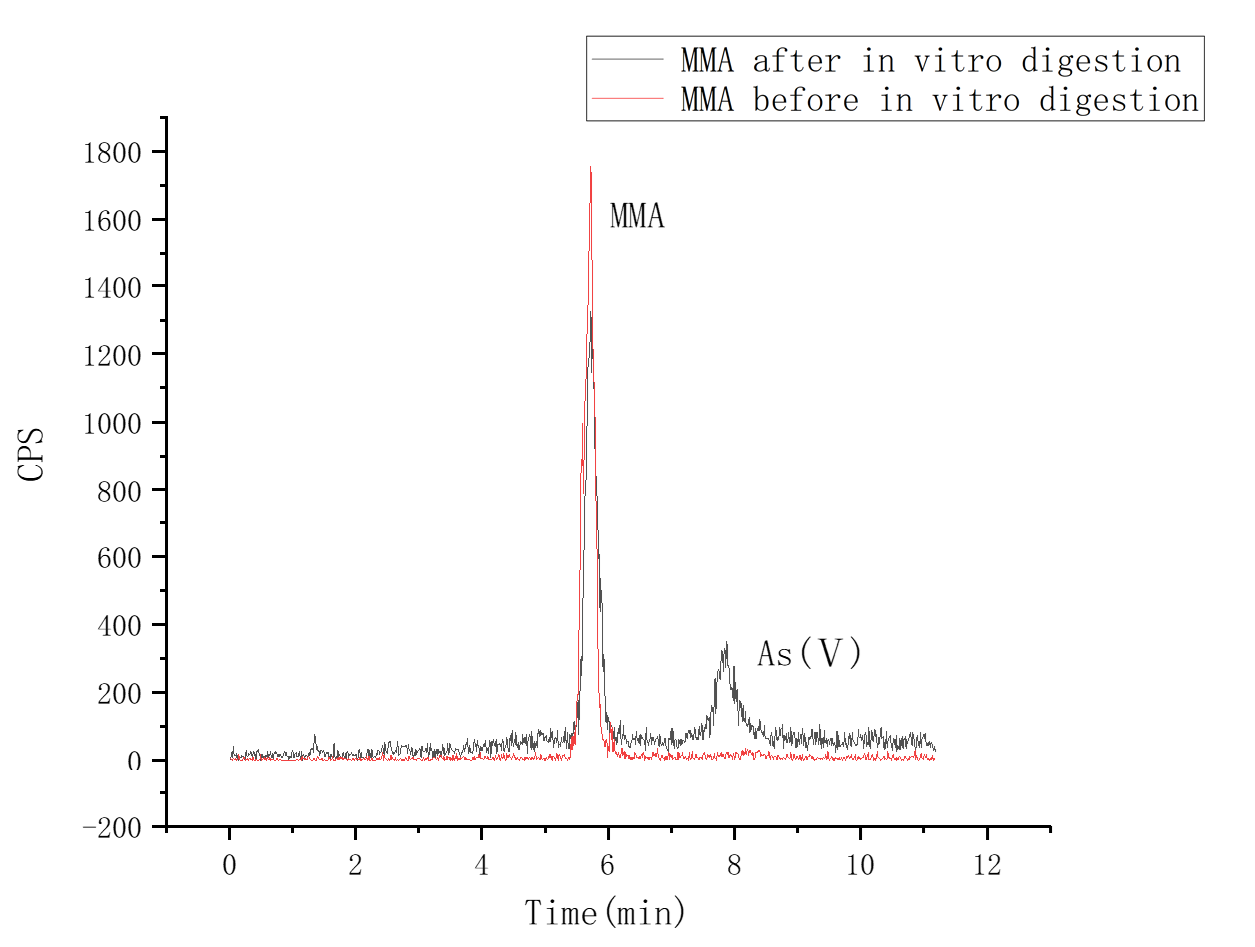


Supplementary Fig. 3. The HPLC-ICP-MS results of the AsC, AsB, DMA, MMA standards before and after *in vitro* digestion.
